# Behavior and Daily Activity Patterns of Specialist and Generalist Predators of the Hemlock Woolly Adelgid, *Adelges tsugae*

**DOI:** 10.1673/031.007.4401

**Published:** 2007-08-14

**Authors:** R.W. Flowers, S.M. Salom, L.T. Kok, D.E. Mullins

**Affiliations:** Department of Entomology, Virginia Polytechnic Institute and State University, Blacksburg, VA, 24061

**Keywords:** *Laricobius nigrinus*, *Sasajiscymnus tsugae*, *Harmonia axyridis*, predator competition, biological control

## Abstract

The behavior and daily activity patterns of two specialist predators, *Laricobius nigrinus* Fender (Coleoptera: Derodontidae) and *Sasajiscymnus tsugae*, Sasaji and McClure (Coleoptera: Coccinellidae), and a generalist predator, *Harmonia axyridis* Pallas (Coleoptera: Coccinellidae), of hemlock woolly adelgid, *Adelges tsugae* (Hemiptera: Adelgidae), were examined using digital video recording in the laboratory. The two specialists are part of a biological control program for *A. tsugae*, and it is not known if competitive interactions with previously established generalist predators will negatively impact their effectiveness. The behavior and daily activity patterns of adult females of each species were documented in single- and paired-predator assays under simulated spring and summer conditions. Behavior varied qualitatively and quantitatively by species, and did not appear to be highly coordinated temporally or spatially. All species exhibited continuous activity patterns that were punctuated by longer periods of rest. Extensive and intensive searching behavior occurred in all species, with intensive searching being highly variable. Specialist predators appeared to be more selective of feeding and oviposition sites, and rested at more concealed locations than the generalist species. In spring conditions, *L. nigrinus* had greater activity and a more even behavior distribution than *S. tsugae* or *H. axyridis*, which were skewed towards resting. In summer, the latter two species showed increased activity at higher temperatures. Conspecifics significantly altered the time allocated to specific behaviors for *L. nigrinus* and *H. axyridis*, resulting in reduced predator effectiveness by reducing time and energy expenditure on activities that directly impact the adelgids. In contrast, *S. tsugae* conspecifics and all heterospecific combinations showed non-interference. The activity of each species varied with time of day; *L. nigrinus* was more active at night, while *S. tsugae* and *H. axyridis* were more active during the day. All predator groupings maintained a high degree of spatial separation relative to assay size. The use of multiple-predator species combinations that include the specialist predators, is recommended over single-species for biological control of *A. tsugae*, as temporal and spatial patterns were not highly coordinated. Low-density releases may reduce the potential negative effects of intraspecific competition.

## Introduction

The hemlock woolly adelgid, *Adelges tsugae* Annand (Hemiptera: Adelgidae), is a major pest of eastern hemlock, *Tsuga canadensis* L. Carriere, and Carolina hemlock, *T. caroliniana* Engelmann, in the eastern United States. This species is believed to originate from southern Japan, where it is an innocuous inhabitant of *Tsuga spp.* (Havill et al. 2006). Populations in these areas are likely regulated by host resistance and natural enemies (McClure et al. 2001). *Adelges tsugae* was first observed in the eastern United States in 1951 in Virginia (Stoetzel 2002), and has since spread south into the southern Appalachians and New England, causing widespread mortality of hemlock species (Cheah et al. 2004). Heavily infested trees exhibit poor crown health and reduced shoot growth that, in combination with other environmental stresses, can result in rapid tree decline and death (McClure 1987; Mayer et al. 2002; Orwig et al. 2002). The tremendous population growth of this species in the eastern United States has been attributed to the absence of effective natural enemies (Cheah and McClure 1998; Wallace and Hain 2000), their ability to survive at low temperatures (Parker et al. 1999), and the high susceptibility of eastern hemlock species.

In North America, *A. tsugae* is anholocyclic, reproducing only asexually on its secondary host, *Tsuga spp*. (McClure 1989). The overwintering generation, or sistens, is present from summer to the following spring (McClure 1989; 1991). Crawlers emerge in April and May, with nymphs developing into wingless and winged forms. The wingless form, or progrediens, remains on hemlock and deposit eggs from which sistens crawlers emerge in June and July, settle at the base of young needles, and immediately enter summer diapause (aestivation) (McClure 1987; Salom et al. 2001). Sistens resume development in October and reach maturity in late winter; thus, two asexual generations are completed annually on hemlock. In ornamental and nursery settings, systemic insecticides have proven effective in managing *A. tsugae* populations (McClure 1992; Rhea 1995; Fidgen et al. 2002; Webb et al. 2003); however, forest hemlocks are found in many inaccessible locations or along riparian zones where chemical controls are neither reasonable nor allowed. Since only generalist pathogens have been identified with *A. tsugae* (Reid et al. 2002) and no known parasitoids are associated with Adelgidae (Montgomery and Lyon 1996), biological control efforts using prey-specific predators have been initiated (Cheah et al. 2004).

*Laricobius nigrinus* Fender (Coleoptera: Derodontidae), native to the western United States and Canada, is a prey-specific predator of *A. tsugae* (Zilahi-Balogh et al. 2002) found on western hemlock *T. heterophylla* (Raf.) Sargent, a species not typically injured by *A. tsugae* (Furniss and Carolin 1977). Adults feed on all stages of *A. tsugae* (Zilahi-Balogh et al. 2002), and show good phenological synchrony with *A. tsugae* (Zilahi-Balogh et al. 2003a). Field studies indicate that *L. nigrinus* significantly reduces *A. tsugae* densities within temporary branch enclosures, and can survive and reproduce in southwest Virginia from November to April (Lamb et al. 2005a; 2006). This species has been mass reared in the laboratory (Lamb et al. 2005b), and released into hemlock stands in the eastern United States since 2003 (Mausel 2004; Lamb et al. 2006).

*Sasajiscymnus* (*Pseudoscymnus*) *tsugae* Sasaji and McClure (Coleoptera: Coccinellidae), native to Japan, is believed to be a specialist predator of *A. tsugae* (Cheah and McClure 1998, Butin et al. 2002). Adults feed on all stages of *A. tsugae* (Sasaji and McClure 1997), and females have a reproductive diapause that coincides with *A. tsugae* aestivation (Cheah and McClure 2000). It is capable of producing successive generations in the laboratory (McClure and Cheah 1999; Palmer and Sheppard 2002). To date, over 1 million beetles have been released into hemlock stands in the eastern United States, and this predator is established within this region (Cheah et al. 2004).

In addition to these newly introduced species, *Harmonia axyridis* Pallas (Coleoptera: Coccinellidae), previously introduced from western Asia for biological control of various hemipteran pests, has quickly spread into many regions of North America (rev. in Koch 2003). Adults are polyphagous, voracious predators (Gordon 1985; Takahashi 1987; Majerus 1994). This species migrates to overwintering sites in late fall (Lamana and Miller 1996; Koch and Hutchinson 2003), and with warmer temperatures in spring, they mate and disperse from these sites (Lamana and Miller 1996). In the southeastern United States, surveys for natural enemies of *A. tsugae* showed that, while predators were generally scarce overall, *H. axyridis* was the most abundant predator collected from branch beat samples (Wallace and Hain 2000).

Although *H. axyridis* is an important arboreal biological control agent of aphids in pecan (Larock and Ellington 1996), apple (Brown and Miller 1998) and citrus (Michaud 2002), its expansion into an area can dramatically affect local populations of aphidophagous predators. Declines in the populations of native, arboreal coccinellids, *Brachiacantha ursine* F., *Cycloneda munda* Say and *Chilocorus stigma* Say in southwestern Michigan (Colunga-Garcia and Gage 1998) and *Cycloneda sanguinea* L. in Florida (Michaud 2002), have been associated with invasion by *H. axyridis*. In addition to impacts on native species, *H. axyridis* has also replaced another established exotic species, *Coccinella septempunctata* L. (Coleoptera: Coccinellidae), as the predominant predator in arboreal habitats in western Oregon (Lamana and Miller 1996) and West Virginia (Brown and Miller 1998). The mechanisms of replacement are not well understood, but direct or indirect competitive interactions among species utilizing the same resource at the same time may lead to reductions in predator diversity and decrease the efficacy of biological control. These interactions can change community structure and ultimately result in the escape of the prey population (Rosenheim et al. 1993), but few studies of competition among predators have been conducted in forest ecosystems.

Beginning in 2002, studies were undertaken to determine the nature and effects of intraspecific and interspecific competition among these species so that the establishment and resilience of newly introduced and existing populations are minimally affected. Both laboratory and field studies by Flowers et al. 2005; 2006 suggest that *L. nigrinus, S. tsugae* and *H. axyridis* are compatible with one another; however, competitive interference was shown to occur among conspecifics. Compatibility was assessed by examining direct impact on the survival, feeding and reproduction of one another when enclosed together in short-term laboratory assays and longer-term field enclosures. However, these studies did not examine more subtle, indirect effects that may have occurred, especially in regard to behavioral changes, which can negatively impact predator effectiveness. Currently, there exist no detailed studies of predator behavior and daily activity patterns of these species with *A. tsugae*, and what type of behavioral changes may occur in the presence of additional predators is unknown. This study was undertaken to determine the daily activity patterns of these species, focusing on extensive and intensive searching, feeding, resting, and oviposition behaviors. Two sets of experimental conditions were used to provide greater coverage over the lifecycle of *A. tsugae* and better approximate the natural conditions under which these interactions may occur. In addition, the effects of intra- and interspecific competition on predator temporal and spatial patterns were examined to more thoroughly examine predator compatibility in this system.

## Materials and Methods

### Insect cultures

*L. nigrinus* adults were obtained from a colony reared at Virginia Polytechnic Institute and State University (Blacksburg, Virginia). *S. tsugae* adults were obtained from colonies at Clemson University (Clemson, South Carolina) and the University of Tennessee (Knoxville, Tennessee). *H. axyridis* adults were collected locally in southwestern Virginia (Jefferson National Forest, Virginia). Laboratory populations of L. *nigrinus* and *H. axyridis* had been supplemented with wild beetles the previous year, while *S. tsugae* were descended from a population that had been consecutively reared for several generations. Predator rearing was in accordance with previously established methods for *L. nigrinus* (10°C, 12:12 L:D, 75% RH) (Lamb et al. 2005b) and the coccinellids (25°C, 16:8 L:D, 45% RH) (Palmer and Sheppard 2002; Matsuka and Niijima 1985). Food adaptation was not a major concern for *L. nigrinus* and *S. tsugae* as they are considered specialists on this prey. The generalist predator, *H. axyridis*, was provided with a small amount of honey and wheast mixture during the holding phase, prior to the experiments, to serve as a nutritional supplement. For the coccinellid species, conditions were gradually stepped down from 20 to 12°C and from 16:8 to 12:12 L:D over a 2-3 wk period to pre-condition them to the conditions used in the spring evaluation. At the conclusion of the spring trials, conditions were gradually increased from 12 to 22°C and from 12:12 to 16:8 L:D over the same time interval to pre-condition predators for summer trials. All species were held in 2.2 L plastic containers lined with moistened filter paper and ventilated with fine polyester mesh (PeCap®, Sefar, www.sefar.com). Each container held 15 adults, in a sex ratio of 2F:1M, and 5–7 *T. canadensis* branch clippings heavily infested with *A. tsugae.* Containers were maintained in an environmental chamber (Model 1–36, Percival-Scientific, www.percival-scientific.com). Adult predators were transferred from holding containers to assays 24 h before recording. All predator females were mature, of approximately the same age (12–24 wk), and were selected randomly from pre-conditioning containers. Morphological characters were used to separate the sex of *S. tsugae* (Sasaji and McClure 1997), and *H. axyridis* (Gordon 1985), while monitoring oviposition over 72 h was used for *L. nigrinus*.

**Figure 1.**
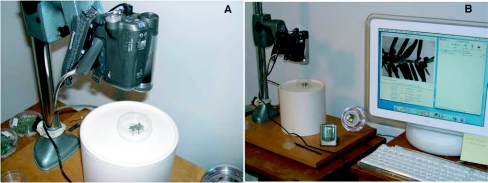
Experimental design for video studies. Each assay consisted of a 5 × 2.5 cm Petri dish lined with moistened filter paper, and held two 5 cm clippings of eastern hemlock, *Tsuga canadensis*, infested with 10 *Adelgus tsugae*. A digital video camera was mounted on a camera stand 5–10 cm above the assay and used for video capture (A). All intermittent video recording was controlled using EvoCam® 3.5 software and video segments were visualized and stored on an Apple iMac® G4 computer (B).

### Experimental design of video studies

Predator behavior and daily activity patterns of adult females of each species were documented in the laboratory using intermittent digital video recording. Predator arenas consisted of 5 × 2.5 cm Petri dishes (Fisherbrand®, Fisher-Scientific, www.fishersci.com) lined with moistened filter paper. The diameter of the assay represented 50-100X the body widths of these predators. Experiments were conducted in an environmentally controlled room using two evaluation periods based on temperature and RH averages obtained by data loggers (Hobo®, Onset Computer, www.onsetcomp.com) placed in hemlock stands in southwestern Virginia. The evaluations were termed 1) spring: 12 ± 2°C, 12:12 L:D, 50–75% RH, and 2) summer: 22 ± 2°C, 16:8 L:D, 65–85% RH. These conditions cover the approximate duration when *A. tsugae* sistens and progrediens adults with ovisacs are present, respectively (McClure 1989). Each assay contained two 5 cm hemlock clippings, which were infested with 10 *A. tsugae* sistens (spring) or progrediens (summer) adults with ovisacs. *A. tsugae* has a clumped distribution at low to moderate densities. These patches have much higher densities than surrounding areas, and the assay was designed to represent such an area. Special attention was paid to prey quantity and quality. Prey quantity was determined from previous laboratory studies (Flowers et al. 2005), such that predators were allowed to feed *ad libitum* during the evaluation. High prey quality was also maintained by microscopically examining areas adjacent to branch clippings used in the trials, to insure that adults were alive and possessed ovisacs with a similar number of eggs. Each predator species was evaluated singly, and combined with one additional conspecific or heterospecific predator in all possible combinations. All species were included in the spring evaluation; however, *L. nignnus* was excluded from the summer evaluation due to its aestivation period (Zilahi-Balogh et al. 2003c).

Video capture was done using a one-chip digital camcorder (PV-GS35, Panasonic, Knoxville, TN) mounted on a camera stand 5–10 cm directly above the arena (Figure 1). The camcorder had a 30X optical lens and 1000X digital zoom. Automatic settings were generally used to control camcorder exposure, gain, focus and white-balance levels; however, manual adjustments were made as necessary to improve overall video quality. A video output cable (FireWire® IEEE 1394, Apple Computer, Cupertino, CA) was used to link the camcorder directly to a computer (iMac® G4, Apple Computer, Cupertino, CA) for direct transfer of all video segments. Digital video software (EvoCam® 3.5, Evological, www.evological.com) was used to visualize the arena, control video-capture and compress video segments prior to storage on the hard drive and eventual archiving onto compact disc. One-minute video recordings were captured every 15 min over 24 h for a total of 96 observations per day. Each treatment was prepared in duplicate, with one assay recorded during the day and one at night. Video segments were compressed to MPEG format and reviewed using QuickTime® 7.0 software (Apple Computer). Two light sources with white translucent diffusing filters were positioned 0.5–1 m on each side of the arena to illuminate the arena adequately for filming. Day recordings used ambient light supplemented by 25W soft-white light bulbs (Philips), while night recordings used 25W red light bulbs (Philips). Pilot studies were completed to determine optimal assay conditions for filming and to assure that predator behavior was not significantly altered in response to experimental conditions. An electronic data logger (Hobo®, Onset Computer) was placed next to the assay to monitor environmental conditions. Six replications of each treatment were completed, with three occurring during the day and three at night.

### Predator behavior and daily activity

The video recordings were reviewed and scored for behavior exhibited and relative location of each predator. Each recording was assigned to one of five general behavior categories: 1) Extensive searching, 2) Intensive searching, 3) Feeding, 4) Oviposition, or 5) Resting. For all species, the duration of each behavior was consistently greater than the 1 min recording interval, so each video segment could be assigned to a single behavioral category. Searching behavior was divided into extensive search, distinguished by rapid movement over a large area of the assay or branch surface, and intensive search, distinguished by slower movements over a small area of the branch surface. Predator placement of the mouthparts, head or body within *A. tsugae* ovisacs was used to designate feeding behavior. This was often accompanied by visual evidence of disrupted woolly filaments and the presence of adelgid hemolymph on the ovisac surface. Close inspection of *A. tsugae* ovisacs, by *L. nigrinus*, or of branch and needle substrates by *S. tsugae* and *H. axyridis*, followed by extension of the posterior abdominal segments (functional ovipositor) was used to designate oviposition behavior. For *L. nigrinus*, egg deposition could not be directly observed because of placement inside the adelgid ovisac. Similarly, egg deposition by the coccinellid species on branch or needle substrates, was difficult to observe directly, as only a single overhead view of the arena was available. Therefore, oviposition events were determined indirectly using microscopic examination of branch clippings at the conclusion of each assay. For all species, each predator egg found was designated as a single oviposition event for that species during that time period. Predator eggs were distinguished using morphological characteristics of *L. nigrinus* (Zilahi-Balogh et al. 2003c), *S. tsugae* (Sasaji and McClure 1997) and *H. axyridis* (Gordon 1985). Resting behavior was designated as predators maintaining a stationary position on the branch or assay surface. Behavior descriptions for each species were based on a review of 15 randomly selected video segments of each behavior category from single-predator assay recordings. Predator daily activity patterns were determined by summing the number of scored events for each behavior category over each time period (day/night). Video segments in which predator behavior could not be clearly distinguished or consisted of multiple behaviors were excluded from the analyses.

### Predator effects on temporal and spatial patterns

In paired-predator assays, behavior was scored for one marked predator, chosen at random, in conspecific combinations and for each species in heterospecific combinations. The impacts of intraand inter-specific competition on predator daily activity patterns were assessed by comparison of the results of single- and paired-predator trials for each species. In the absence of significant competitive interference, similarity among the counts of each behavior category would be expected among the treatments. A significant shift in the number of behavior events was used to indicate predator interference. For spatial analyses of predator pairs, the approximate separation (0–5 cm) between predators was assessed at the start of each recording, and distances were averaged for each treatment. Separation distances were measured from the center of the body of each predator to account for size differences among these species.

### Statistical analysis

Predator behavior analyses as well as changes in temporal and spatial activity patterns were examined separately for the spring and summer evaluation using a mixed model (proc mixed;SAS Institute 2001, www.sas.com). The model incorporated both fixed factors and a random covariate, as behavior was documented for individual predators and required a hierarchical analysis since the individual served as a block for the behaviors. Single-predator assay comparisons were analyzed separately by behavior category and the model included predator species and time (day/night) as fixed categorical variables and predator species by replication as the random covariate. Paired-predator temporal behavioral analyses were examined separately by predator species and behavior category, while paired-predator spatial analyses were examined separately by behavior category only. The model in each case included predator species combination and time as fixed categorical variables and predator species combination by replication as the random covariate. Treatment means were analyzed using two-way analysis of variance followed by Tukey's HSD to separate treatment means (Zar 1998). Behavior event counts were log10 transformed to achieve normality and equality of variances, and all results were evaluated for significance at *P* ≤ 0.05.

## Results

### Predator behavior

Adult female predator behavior varied qualitatively for each species. Extensive searching behavior was similar in form for all species. It occurred primarily on the upper and lower assay surfaces and consisted of successive rapid movements covering a large area. The behavior was sometimes followed by relocation onto branch surfaces, and a rapid shift to intensive searching behavior occurred. In other cases, the behavior would occur over longer periods of time, and may represent predator agitation and an attempt to disperse from the assay. Intensive searching by *L. nigrinus* consisted of successive inspection of *A. tsugae* ovisacs using a series of short linear movements from one prey location to another (Video). During prey inspection, there was often tapping of the antennae and abdomen on the surface of the adelgid ovisac. After surveying numerous prey items, adult females would remain at a single location for additional inspection, and this was often followed by feeding or oviposition behavior. Intensive searching by *S. tsugae* consisted of a series of systematic movements along the needle and branch surfaces. In most cases, one side of the branch clipping would be surveyed at a time by horizontal movements along each side of several needles in succession (Video). This often resulted in contact Video. This video can be accessed at the following URI: http://digital.library.wisc.edu/1793/11389 with *A. tsugae* ovisacs, after which this search pattern would cease and an apparent closer inspection of prey would occur. For *H. axyridis*, intensive searching appeared to be less systematic than that of the specialist predators. Females would usually move from an extensive searching pattern or a resting location directly to a prey in close proximity. Prey selection was rapid and feeding often began immediately.

Feeding by adult female predators was similar in form for all species. The mandibles were used to disrupt the ovisac filaments to obtain access to adult and immature *A. tsugae*. Specialist predators would usually feed on some of the outer eggs of the adelgid ovisac, then advance further into the ovisac until only the posterior abdomen was visible, causing a low level of disruption (Video). When attacking adelgid adults, specialist predators would often make a single bite on the thorax or abdomen, releasing hemolymph that was ingested by the adult, and resulting in a collapsed *A. tsugae* exoskeleton. In contrast, *H. axyridis* would greatly disrupt adelgid ovisacs to feed on adults and immatures, often consuming a large portion of the eggs and adult exoskeleton by chewing (Video). Feeding by the specialists occurred more often and for shorter durations than *H. axyridis.*

Predator oviposition and resting behavior for these species was similar in form and distinct in location. For *L. nigrinus*, there were tapping and repeated insertions of the posterior abdominal segments into the adelgid ovisac before egg deposition (Video). Lateral movements of the legs and abdomen helped to drive these segments deeper into the ovisac. For this species, eggs were usually placed singly, but 2–3 eggs would sometimes be placed at the same location once all available ovisacs contained eggs. Oviposition by *S. tsugae* and *H. axyridis* was very low overall, and difficult to document in many cases due to experimental limitations. It appeared that the these coccinellid species would first inspect the foliage or branch surface, followed by extension of the posterior abdominal segments, which would probe host substrates before oviposition. For *S. tsugae*, eggs were usually placed singly in close proximity to adelgid ovisacs on hemlock bud scales or in bark crevices with the eggs slightly exposed. In contrast, oviposition by *H. axyridis* was in groups of 5–15 eggs placed on needle surfaces in close proximity to adelgid ovisacs. Lastly, resting behavior by *L. nigrinus* and *S. tsugae* usually occurred at concealed locations on the branch surface in close proximity to *A. tsugae*. Predators were often located at the junction of the stem and a branchlet or between two needles at the base of branchlets. In contrast, resting by *H. axyridis* occurred at more exposed locations on branch and arena substrates often at greater distances from *A. tsugae*.

**Figure 2.**
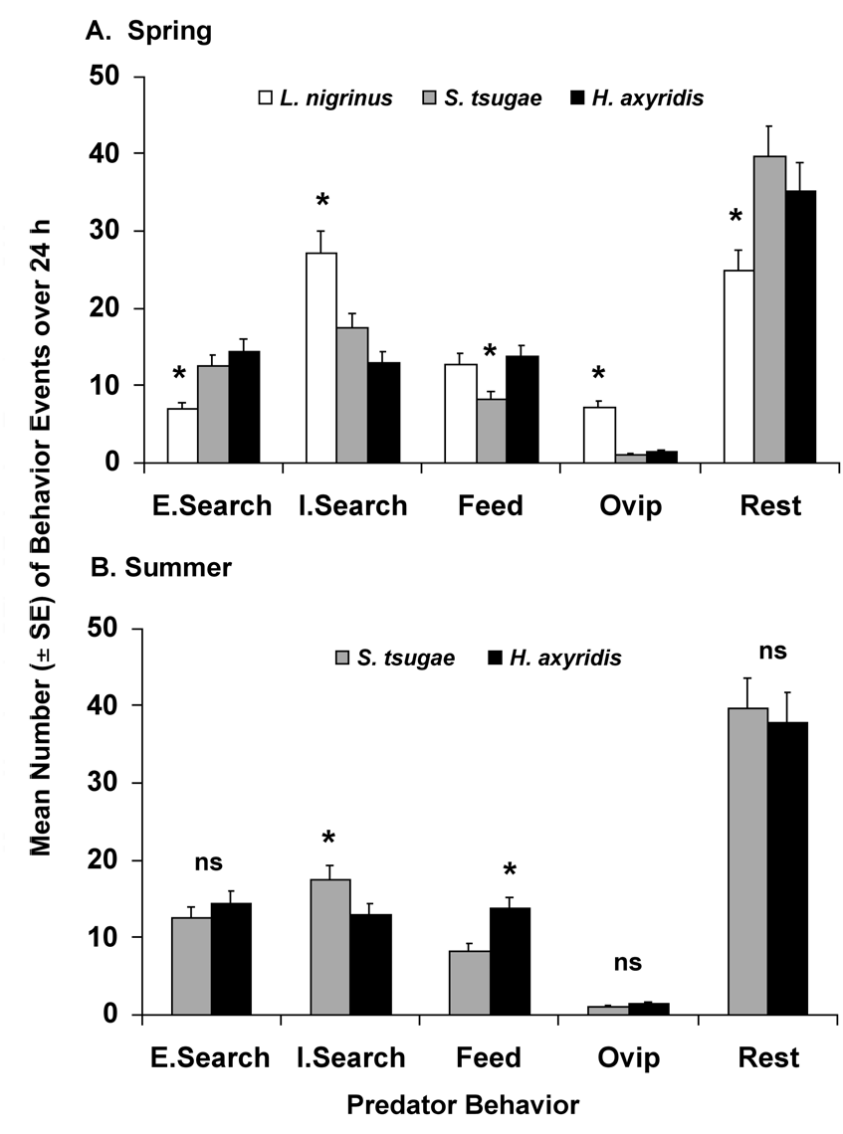
Mean number (± SE) of behavior events per day for adult female *Laricobius nigrinus, Sasajiscymnus tsugae* and *Harmonia axyridis* grouped into five behavior categories (Extensive Searching, Intensive Searching, Feeding, Resting, Oviposition) during (A) spring (12±2°C, 12:12 L:D, 50–75% RH) and (B) summer (22±2°C, 16:8 L:D, 65–85% RH) evaluations in single-predator assays (n=6). One-minute video segments were captured every 15 min over 24 h. ^*^Mean was significantly different at *P* ≤0.05, ns = no significant difference among means for the behavior.

**Figure 3.**
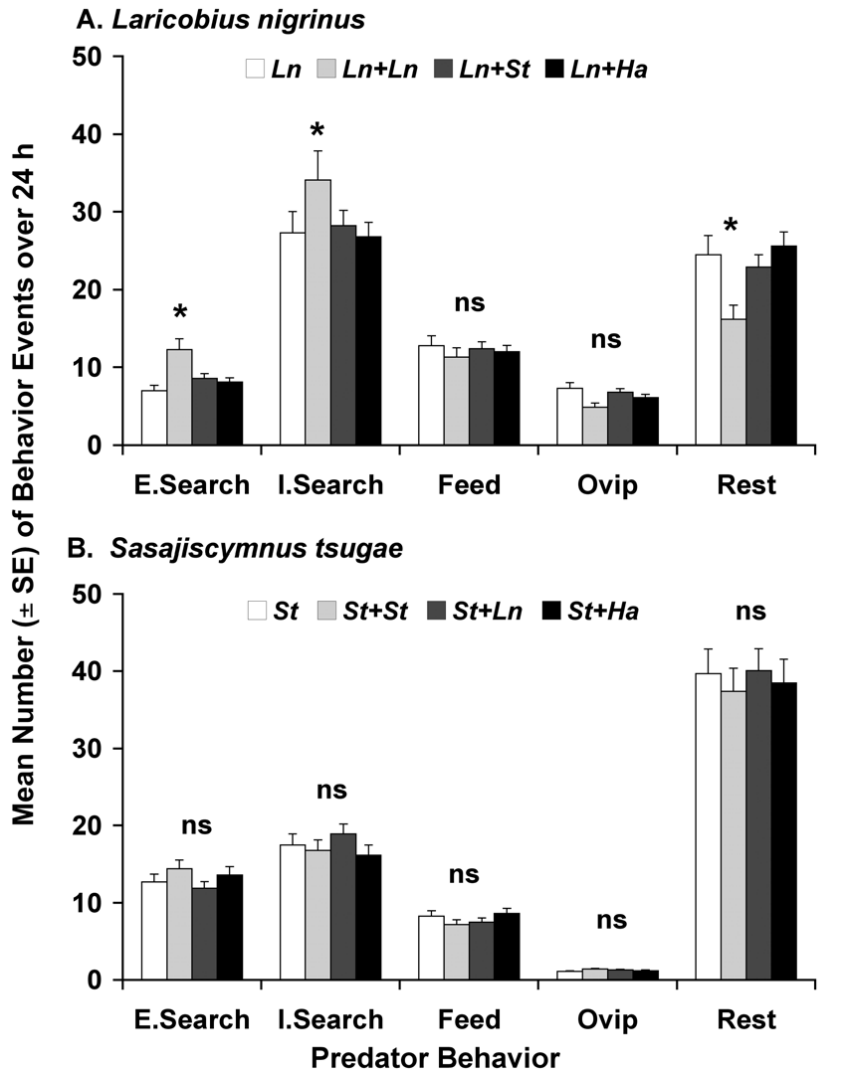
Mean number (± SE) of behavior events per day for adult female (A) *Laricobius nigrinus* (*Ln*), (B) *Sasajiscymnus tsugae* (*St*) and (C) *Harmonia axyridis* (*Ha*) grouped into five behavior categories (Extensive Searching, Intensive Searching, Feeding, Resting, Oviposition) during the spring evaluation (12±2°C, 12:12 L:D, 50–75% RH) in paired-predator assays (n=6). Each species was tested alone and in combination with one conspecific or heterospecific predator. One-minute video segments were captured every 15 min over 24 h. Analyses were done separately for each behavior. ^*^Mean was significantly different at *P* ≤ 0.05, ns = no significant difference among means for the behavior, Each species was tested alone and in combination with one conspecific or heterospecific predator. One-minute video segments were captured every 15 min over 24 h. Analyses were done separately for each behavior. ^*^Mean was significantly different at *P* ≤ 0.05, ns = no significant difference among means for the behavior.

**Continue:**
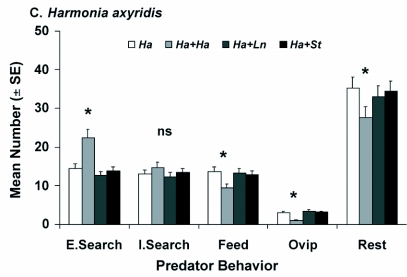


### Predator daily activity

In single-predator behavior comparisons, there was a significant interaction between predator species and behavior category in spring (*F* = 5.34; df = 8,54; *P* ≤ 0.0001) and summer (*F* = 4.63; df = 4,36; *P* ≤ 0.0001), so each behavior category was analyzed separately. For the behavior category analyses, there were no significant interactions between the variables, so each factor was evaluated across the other variable levels.

During the spring, there were significant differences by predator species for extensive searching, intensive searching, feeding, oviposition and resting (Figure 2A, Table 1). Extensive searching and resting behaviors were greater in the coccinellid species than in *L. nigrinus*, which showed greater intensive searching behavior and oviposition than the coccinellid species. Feeding behavior by *L. nigrinus* and *H. axyridis* was similar in the spring, and counts for each species were greater than for *S. tsugae*. Overall, the distribution of events across behavior categories was more even for *L. nigrinus*, while the coccinellid behaviors were skewed toward resting.

**Table 1.**
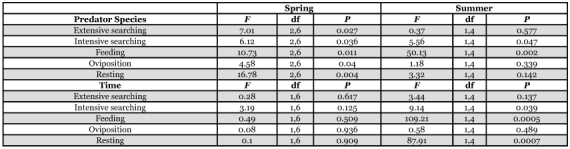
Daily activity comparisons of single predators by species and time (day/night).

During the summer evaluation, significant differences also occurred by predator species for intensive searching and feeding, while extensive searching, oviposition and resting were similar (Figure 2B, Table 1). Intensive searching behavior was greater for *S. tsugae* than for *H. axyridis*. The latter had a greater number of feeding events than *S. tsugae*. During the summer evaluation, behavior for these species appeared to be more evenly distributed across the categories than in the spring.

For the second factor, time (day/night), there were no significant differences in the spring for extensive searching, intensive searching, feeding, oviposition and resting, when all predator species responses were analyzed together. In contrast, during the summer, there were significant differences in intensive searching, feeding and resting, while extensive searching and oviposition were similar (Table 1). During this period, intensive searching and feeding behavior for the coccinellids were greater during the day while resting was greater at night.

**Figure 4.**
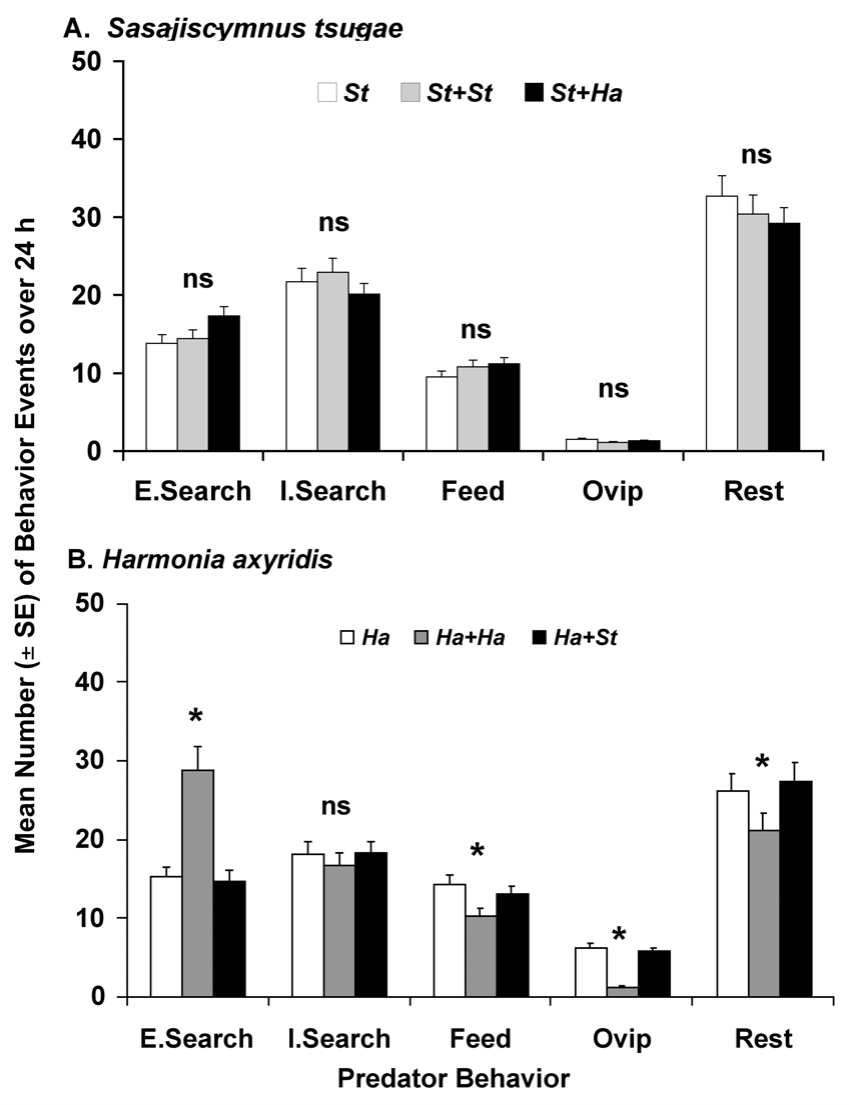
Mean number (± SE) of behavior events per day for adult female (A) *Sasajiscymnus tsugae* (*St*) and (B) *Harmonia axyridis* (*Ha*) grouped into five behavior categories (Extensive Searching, Intensive Searching, Feeding, Resting, Oviposition) during the summer evaluation (22±2°C, 16:8 L:D, 65–85% RH) in paired-predator assays (n=6). Each species was tested alone and in combination with one conspecific or heterospecific predator. One-minute video segments were captured every 15 min over 24 h. Analyses were done separately for each behavior. ^*^Mean was significantly different at *P* ≤ 0.05, ns = no significant difference among means for the behavior.

**Table 2.**
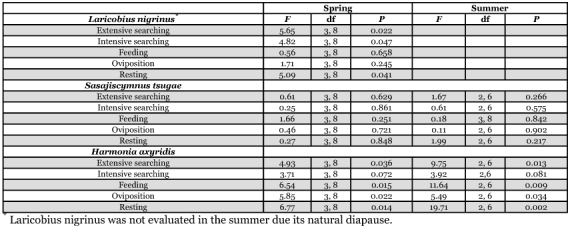
Predator effects on temporal patterns by species in paired-predator trials.

### Predator effects on temporal patterns

For temporal activity comparisons in the paired-predator trials, there were significant interactions between predator species combination and behavior category during the spring (F = 4.28; df = 12,72; P ≤ 0.0001) and summer (F = 2.28; df = 8,54; P = 0.003), so behavior categories were analyzed separately. For each behavior category, there were no significant interactions between the variables, so each factor was evaluated across the other variable levels.

In the spring, *L. nigrinus* showed significant differences by predator species combination for extensive searching, intensive searching, and resting, while feeding and oviposition were similar (Figure 3A, Table 2). For each behavior, it was the conspecific assay that varied, leading to greater extensive and intensive searching behaviors and decreased resting. In contrast, *S. tsugae* had similar responses by predator species combination for all behaviors (Figure 3B, Table 2). For *H. axyridis* in the spring, there were significant differences by predator species combination for extensive searching, feeding, oviposition, and resting, while intensive searching was similar (Figure 3C, Table 2). As in the case for *L. nigrinus*, it was the conspecific treatment that varied, leading to more extensive searching, but reduced feeding, oviposition and resting.

**Table 3.**
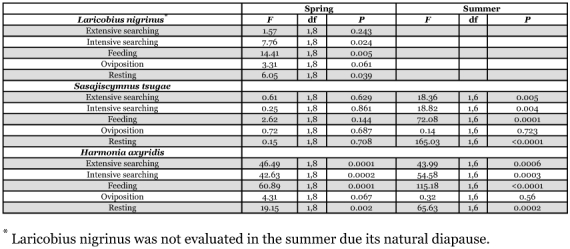
Predator effects on temporal patterns by time(day/night) in paired-predator trials.

The coccinellid predators showed similar results during the summer with *S. tsugae* showing no significant differences by predator species combination for all behaviors (Figure 4A, Table 2). In contrast, *H. axyridis* again showed significant differences by predator species combination for extensive searching, feeding, oviposition and resting, while intensive searching was similar (Figure 4B, Table 2). Consistent with the spring evaluation, the conspecific combination led to increased extensive searching as well as reduced feeding, oviposition, and resting.

**Figure 5.**
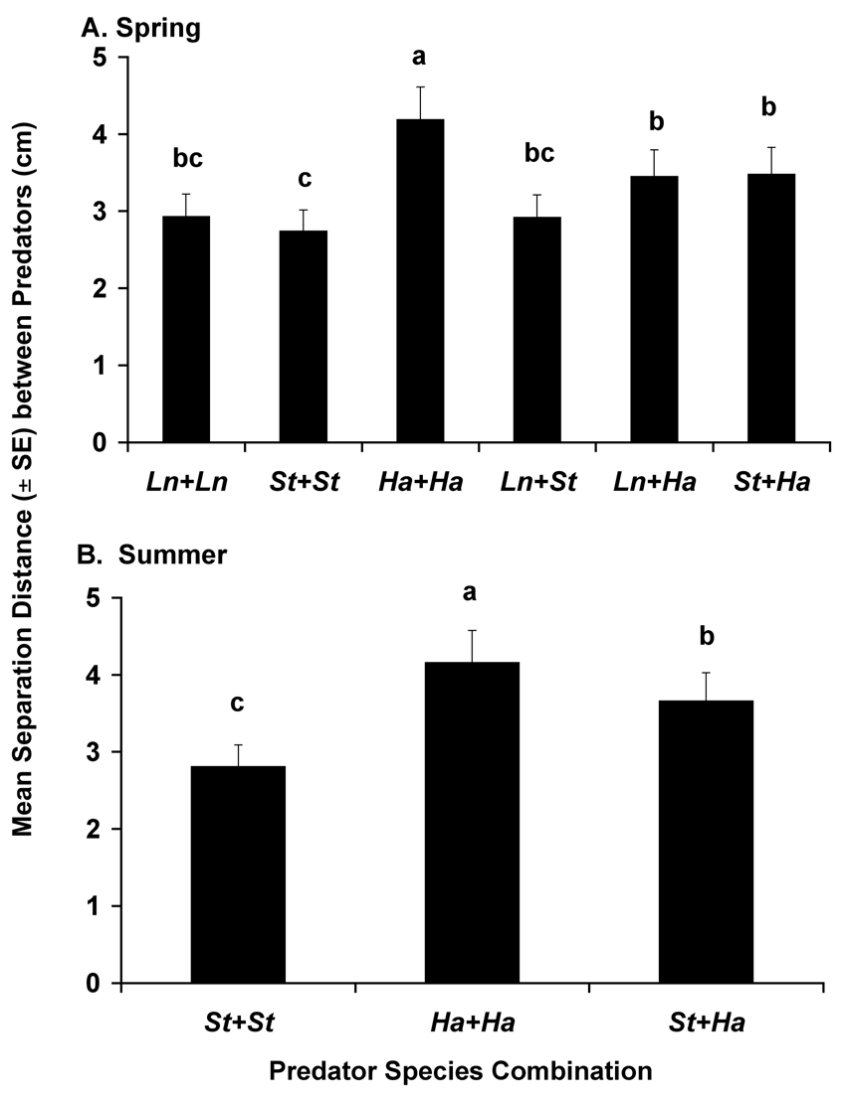
Mean separation distance (± SE) between adult female *Laricobius nigrinus* (*Ln*), *Sasajiscymnus tsugae* (*St*) and *Harmonia axyridis* (*Ha*) in the (A) spring (12±2°C, 12:12 L:D, 50–75% RH) and (B) summer (22±2°C, 16:8 L:D and 65–85% RH) evaluations for the paired-predator assays (n=6). Each species was tested in combination with one conspecific or heterospecific predator. One-minute video segments were captured every 15 min over 24 h. Means with the same letter were not significantly different at P ≥ 0.05.

By time (day/night), the spring evaluation revealed significant differences for *L. nigrinus* in intensive searching, feeding and resting, while extensive searching and oviposition were similar (Table 3). There was greater intensive searching behavior at night, while feeding and resting were greater during the day. For *S. tsugae*, no significant differences by time were observed for any of the behaviors in the spring (Table 3). The results for *H. axyridis* revealed significant differences by time for extensive searching, intensive searching, feeding and resting, while only oviposition was similar (Table 3). Extensive and intensive searching, as well as feeding, were greater during the day, while resting occurred more at night.

In the summer, *S. tsugae* showed significant differences by time for extensive and intensive searching, as well as feeding and resting, while only oviposition was similar (Table 3). Results were similar for *H. axyridis*, with extensive searching, intensive searching, feeding and resting significantly different by time, while oviposition was similar (Table 3). For both coccinellid species, extensive and intensive searching, as well as feeding, was greater during the day, while resting events were greater at night.

### Predator effects on spatial patterns

Spatial activity comparisons in the paired-predator trials showed no significant interactions between predator species combination and time during spring (F = 1.67; df = 5,12; P = 0.218) or summer (F = 0.46; df = 2,6; P = 0.651), and each factor was evaluated across all other variable levels. There were significant differences by predator species combination during both spring (F= 7.46; df = 5,12; P = 0.002) (Figure 5A) and summer (F= 23.27; df = 2,6; P = 0.001) (Figure 5B). In the spring, the conspecific pairing of *H. axyridis* showed the greatest average separation distance, followed by *L. nigrinus* conspecifics and all heterospecific pairings, which were similar. During the summer evaluation, *H. axyridis* conspecifics once again maintained the greatest spatial separation, followed by the heterospecific pairing. The conspecific pairing of *S. tsugae* were found in closest proximity during each evaluation. Predator separation distances were not significantly different by time during the spring (F = 4.19; df = 2,12; P = 0.068) or summer (F = 0.52; df = 1,6; P = 0.497) evaluation.

## Discussion

Within-habitat host searching by aphidophagous predators does not usually occur randomly, but involves two patterns of movement. Extensive searching uses rapid linear movements between prey patches, while intensive searching includes slower and more directed movements that are often induced by the perception of prey cues (Bell 1990). In predatory beetles, changes to search behavior often occur in response to prey detection and capture, which results in a switch from extensive to intensive, or local area-concentrated, searching (Nakamuta 1982; 1985). This varies based on the ability of the predators to detect prey-specific cues, and appears to be advantageous for predators whose prey display a clumped distribution. Previous studies of *H. axyridis* indicate that this species uses vision and olfaction to find prey (Obata 1997; Harmon et al. 1998; Han and Chen 2002), and are consistent with the observations presented here with A. tsugae. Adults of *H. axyridis*, unlike the specialist predators, did not appear to follow any type of systematic search pattern to find prey, but consistently demonstrated the ability to move directly to prey locations under both day and night conditions. While specific cues were not directly tested in this study, the behavioral observations are suggestive of their use. Olfaction may also be used in prey finding by *L. nigrinus*, which possess antennae with several olfactory receptors (Broeckling and Salom 2003), and may be able to detect *A. tsugae* volatiles. Field studies indicate that *L. nigrinus* can locate *A. tsugae* at low population densities in its native range (Mausel 2005), suggesting that it may have long-range prey perception ability. In contrast, *S. tsugae* has a less sophisticated antennal morphology, with few olfactory receptors (Broeckling and Salom 2003), and thus may rely more on vision and direct contact cues to locate prey, as was observed in this study.

The differences in intensive searching behavior in these species are likely related to predator feeding and reproductive biology. For *L. nigrinus* and *S. tsugae*, greater prey evaluation may be a product of their close association with A. tsugae (Zilahi-Balogh et al. 2002; Cheah and McClure 1998). Eggs of *L. nigrinus* are laid directly within adelgid ovisacs, and thus, it would be advantageous for females to insure that each ovisac is of sufficient size or quality for the successful development of progeny. Similarly, rearing studies indicate that *S. tsugae* is sensitive to prey quality (Palmer and Sheppard 2002), which may explain its more thorough prey evaluation once A. tsugae was located. In contrast, *H. axyridis* may not be adapted to detect qualitative differences of A. tsugae, and intensive searching in this species appears to be directed toward areas of high prey density that are within close proximity. These results are consistent with previously described area-concentrated searching behavior for *Coccinella septempunctata* L., a closely related coccinellid predator (Nakamuta 1985; 1987) and for *H. axyridis* with aphid prey in Japan (Obata 1986; Osawa 2000). While the former species used primarily visual cues, a combination of visual and olfactory cues appears to be used by *H. axyridis* to guide movements to prey.

Variations in feeding and oviposition behaviors are also consistent with what is known regarding predator foraging and reproductive biology. Feeding and oviposition behaviors by *L. nigrinus* and *S. tsugae* were consistent with what has been described previously for another specialist predator of A. tsugae (Lu et al. 2002). Microscopic examinations of assays confirmed that feeding events by *L. nigrinus* and *S. tsugae* were often limited in extent. Feeding that is less disruptive to the ovisac may be advantageous for *L. nigrinus*, as it may provide increased protection for progeny that develop within the adelgid ovisac. In contrast, feeding by *H. axyridis* was generally more extensive at each site and resulted in a greater disruption to ovisac filaments. This in turn may serve to better facilitate entry by progeny that emerge from eggs placed on host substrates in close proximity. The ovipositional behavior observed for *L. nigrinus* may serve to provide an additional assessment of prey quality or potentially assist in the detection of conspecific chemical markers or conspecific eggs. This could allow *L. nigrinus* to reduce intraspecific interactions among progeny, which is detrimental to larval survival. A similar type of assessment, of branch and needle substrates, also occurred for the coccinellid species before oviposition. These behaviors may serve to discriminate locations of better attachment or protection of eggs, and could lead to increased progeny survivorship.

Predator oviposition locations may be related to reducing interspecific competition, as eggs of *L. nigrinus* and *S. tsugae* are vulnerable to predation by all three species (Flowers et al. 2005; 2006). Thus, placement at more concealed locations lowers the risk of predation or provides a higher degree of buffering against environmental conditions. In contrast, the visibility of *H. axyridis* eggs appears to be related to their inherent chemical deterrents, which provide protection against interspecific predation (Ayer and Brown 1977; Hemptinne et al. 2000; Agarwala and Yasuda 2001). In addition, the bright coloration and clumped distribution of eggs may serve to warn heterospecifics, and provide increased detection and food availability to conspecifics. When prey is of low quantity or quality, cannibalism has been shown to increase progeny growth and survival (Wagner et al. 1999; Snyder et al. 2000; Michaud and Grant 2004).

Variations in resting behavior followed a similar pattern, with *L. nigrinus* and *S. tsugae* being located in more concealed locations and *H. axyridis* maintaining greater apparency. Generalist predators such as *H. axyridis* often engage in intraguild predation when prey becomes scarce (Yasuda and Shinya 1997; Burgio et al. 2002; 2005); therefore, more concealed resting locations may provide additional protection for the specialist species. Greater apparency for *H. axyridis* would not be detrimental to their survival given their chemical ecology and warning coloration, and may serve to increase mate-finding (Majerus 1994).

Daily predator activity for these species can be generally classified as continuous, in which shorter searching, feeding and oviposition events are punctuated by longer periods of rest. In the spring, increased activity and a more even behavior distribution by *L. nigrinus* was likely due to this species being highly cold-adapted (Lamb et al. 2005a; Zilahi-Balogh et al. 2003b). Behavior in these species is influenced by temperature (Cheah and McClure 1998; Stathas et al. 2001; Zilahi-Balogh et al. 2002); the developmental threshold of *L. nigrinus*, which is lower than *S. tsugae* or *H. axyridis* by 5.8 and 7.5°C, respectively (Cheah and McClure 1998; Lamana and Miller 1998; Zilahi-Balogh *et al*. 2003c), allowed this species to maintain greater activity levels under colder conditions. Consistent with this result, *S. tsugae* and *H. axyridis* appeared to have a more even behavioral distribution at higher temperatures in the summer evaluation. However, even when temperatures were similar to optimal rearing conditions for the coccinellid species, oviposition may still have been limited due to experimental constraints such as insufficient prey quality and host health for *S. tsugae* (Palmer and Sheppard 2002) and nutrient limitations for *H. axyridis* (Dixon 2000; Scares et al. 2004). In addition, the design may also have limited the dispersal ability of these species to potentially more favorable microhabitats.

Conspecific predators exerted influence on the behavior patterns of *L. nigrinus* and *H. axyridis*, but not of *S. tsugae*. In the spring, the addition of *L. nigrinus* conspecifics increased searching behaviors and decreased resting, while feeding and oviposition remained similar. This response is most likely related to prey selectivity, as females had to spend more time locating acceptable oviposition sites. Predator effectiveness, in terms of energy expenditure, would therefore be reduced, as *L. nigrinus* dedicated more time and resources to searching behaviors, which do not serve to directly impact adelgid prey. If behaviors in this species were altered in response to passive chemical cues associated with conspecific contact of prey or branch substrates, we would expect feeding and oviposition rates to be affected also; however, these responses were not affected. The results also indicate that females of this species may be adapted to detect the presence of eggs or oviposition-deterring chemical cues of conspecifics. The wide-ranging effects of conspecifics on *H. axyridis* behavior are consistent with previous studies of the chemical ecology of this predator. Intraspecific interactions in this species are regulated by both passive substrate marking using fecal cues (Agarwala et al. 2003) and actively deposited oviposition-deterring pheromones (Yasuda et al. 2000). Given the temporary nature of many prey populations and its ability to cannibalize (Osawa 1993; Hironori and Katsuhiro 1997; Burgio et al. 2002; 2005), selective pressure would favor avoiding or reducing activity in areas already occupied by conspecifics. This was consistent with the results observed in these trials where extensive searching was increased, and feeding and oviposition were reduced. Conspecific effects for *S. tsugae* may have been masked by the predominance of resting behavior in these trials, as previous studies indicated that this species has the ability to cannibalize (McClure 1995), and its behavior may therefore be regulated by similar mechanisms. In contrast, predator temporal activity patterns were not significantly altered by heterospecifics, indicating that these species may be compatible in this system and suggesting that these predators may not be adapted to detect the chemical cues of one another.

Each species displayed distinct temporal patterns in which behaviors occurred more frequently during day or night. For *L. nigrinus*, greater intensive searching at night and resting during the day may indicate this species to be more nocturnal than the other two predators. Increased behavior at this time is possible because of adaptation to cold (Zilahi-Balogh *et al* 2003a), and may serve to decrease susceptibility to predators or reduce co occurrence with other species. Searching behavior during this time also suggests that it may rely more on olfaction than vision to locate and evaluate prey. In contrast, *S. tsugae* and *H. axyridis* were more active during the day, consistent with previous observations of other coccinellid predators (Nakamuta 1987; Majerus 1994) and *H. axyridis* (Obata and Johki 1990). Greater searching and feeding activity during the day may be related in some degree to increased daytime temperatures, but more likely, this may have resulted from their using visual cues to locate *A. tsugae*. To optimize finding prey and conserve energy and resources, search activity in these species may be reduced at night due to a lower success of prey detection in total darkness. Oviposition by the coccinellids would likely have been statistically higher during the day as well due to similar factors, but overall low levels of reproduction make these inferences difficult.

Relative to assay size, all predator pairings appeared to maintain a high degree of spatial separation. While it is difficult to assess the responses due to the limited size and architecture of the assay, this suggests that avoidance behaviors may occur in these species. Distances were greatest between conspecifics of *H. axyridis* and this may again be in response to chemical cues, as has been documented in spatial studies of similar predator species (Grostal and Dicke 1999). The shorter separation distances for *L. nigrinus* and *S. tsugae* conspecifics, by comparison, may indicate these species have a less sophisticated chemical ecology. For *S. tsugae*, distance measures were influenced by the predominance of resting behavior, during which conspecifics were found in close proximity to one another. However, separation between *S. tsugae* conspecifics during searching and feeding events was similar to that of the other species. Heterospecific pairings also exhibited high spatial separation within the context of these assays. Overall, interspecific effects on spatial patterns appeared be in response to tactile (direct contact), rather than chemical cues. Video evidence consistently showed that predator disturbance and re-location was high in response to direct contact, particularly by the specialists when contacted by *H. axyridis*. Avoidance behaviors and changes in spatial patterns in response to heterospecifics have been shown to occur in other aphidophagous predator guilds (Musser and Shelton 2003; Sato et al. 2005), and may regulate spatial relationships in this system as well.

Inferences based on these observations are limited due to the experimental design and duration, which may have exerted a great deal of influence on the behavior of these species. The evaluation of extensive search behavior was perhaps most limited, as predators were prohibited from leaving the arena; however, the movement pattern exhibited within the arena was suggestive of extensive search behavior. Consanguinity should also be considered, as laboratory populations may not accurately represent the behavioral patterns that occur within the larger population. However, *L. nigrinus* and *H. axyridis* colonies were regularly supplemented with wild beetles collected in the field. For *S. tsugae*, adults were descended from a limited, original collection and have been consecutively reared for several generations, and thus may have reduced behavioral variability. Predators have also found to display a high degree of plasticity in individual behaviors, and previous studies indicate that searching patterns can change in response to prey type, and that behavior may be conditioned in some beetle species with continuous exposure to a single prey (Ettifouri and Ferran 1993). Also, the techniques necessary to study predator behavior in detail do not allow for the immigration and emigration that may occur on a larger scale in response to prey abundance or intraspecific and interspecific predator cues. Video recordings of predator behavior were difficult to obtain using a single camcorder due to the architecture of the hemlock clippings, which often shielded predator activity from view. In addition, video quality was limited by using a standard camcorder, and a more sophisticated design using a camera with greater specialization may assist in better documenting individual behaviors in these species. However, this experimental model provided an inexpensive and highly flexible method to conduct video studies of behavior, and could be implemented in a variety of ways to conduct these types of analyses.

In conclusion, additional examinations of these predators under field conditions are necessary to corroborate descriptions of predator behavior and daily activity. However, activity patterns for *H. axyridis*, documented in field studies in Japan (Obata and Johki 1990), were very similar to those found for this species using the laboratory techniques presented here. These behavioral analyses support our previous studies that indicate these species to be compatible within a biological control program for A. tsugae. Temporal activity patterns were not highly coordinated, and predator avoidance responses appear to be such that these species will be able to maintain a high degree of spatial separation under more natural conditions. Therefore, we recommend using multiple-predator combinations of the specialist predators, over single-species when implementing biological control for A. tsugae. In addition, implementing low-density predator releases may reduce the potential negative effects associated with intraspecific interference and lead to improved predator effectiveness under field conditions.

## References

[bibr01] Agarwala BK Yasuda H. 2001 Overlapping oviposition and chemical defense of eggs in two co-occurring species of ladybird predators of aphids. *Journal of Ethology* 19 47 53

[bibr02] Agarwala BK Yasuda H Kajita Y. 2003 Effect of conspecific and heterospecific feces on foraging and oviposition of two predatory ladybirds: Role of fecal cues in predator avoidance. *Journal of Chemical Ecology* 29 357 376 12737263 10.1023/a:1022681928142

[bibr03] Ayer WA Brown LM 1977 The ladybug alkaloids including synthesis and biosynthesis. *Heterocycles* 7 685 707

[bibr04] Bell WJ 1990 Searching behavior patterns in insects. *Annual Review of Entomology* 35 447 467

[bibr05] Broeckling CD Salom SM 2003 Antennal morphology of two specialist predators of hemlock woolly adelgid, *Adelges tsugae* Annand (Homoptera: Adelgidae). *Annals of the Entomological Society of America* 96 153 160

[bibr06] Brown MW Miller SS 1998 Coccinellidae (Coleoptera) in apple orchards of eastern West Virginia and the impact of invasion by *Harmonia axyridis*. *Entomological News* 109 143 151

[bibr07] Burgio G Santi F Maini S 2002 On intra-guild predation and cannibalism in *Harmonia axyridis* (Pallas) and *Adalia bipunctata* L. (Coleoptera: Coccinellidae). *Biological Control* 24 110 116

[bibr08] Burgio G Santi F Maini S 2005 Intra-guild predation and cannibalism between *Harmonia axyridis* and *Adalia bipunctata* adults and larvae: laboratory experiments. *Bulletin of Insectology* 58 135 140

[bibr09] Butin E Montgomery M Havill N Elkinton J 2002 Pre-release host range assessment for classical biological controls: Experience with predators for hemlock woolly adelgid. Onken B Reardon R Lashomb J *Proceedings: Hemlock Woolly Adelgid in the Eastern United States Symposium* 205 213 Rutgers University

[bibr10] Cheah CASJ McClure MS 1998 Life history and development of *Pseudoscymnus tsugae* (Coleoptera: Coccinellidae), a new predator of the hemlock wooly adelgid (Homoptera: Adelgidae). *Environmental Entomology* 27 1531 1536

[bibr11] Cheah CASJ McClure MS 2000 Seasonal synchrony of life cycles between the exotic predator, *Pseudoscymnus tsugae* (Coleoptera: Coccinellidae) and its prey, hemlock woolly adelgid *Adelges tsugae* (Homoptera: Adelgidae). *Agricultural and Forest Entomology* 2 241 251

[bibr12] Cheah CASJ Montgomery ME Salom SM Parker B Costa S Skinner M 2004 Onken B Reardon R *Biological control of hemlock woolly adelgid* 1 22 USDA Forest Service

[bibr13] Colunga-Garcia M Gage SH 1998 Arrival, establishment, and habitat use of the multicolored Asian lady beetle (Coleoptera: Coccinellidae) in a Michigan landscape. *Environmental Entomology* 27 1574 1580

[bibr14] Dixon AFG 2000 *Insect predator-prey dynamics: Ladybird beetles and biological control.* Cambridge University Press

[bibr15] Ettifouri M Ferran A 1993 Influence of larval rearing diet on the intensive searching behavior of *Harmonia axyridis* (Col.: Coccinellidae) larvae. *Entomophaga* 38 51 59

[bibr16] Fidgen JG McClellan QC Salom SM 2002 Efficacy and residual activity of two systemic insecticides for control of hemlock woolly adelgid on young eastern hemlocks. Onken B Reardon R Lashomb J *Proceedings: Hemlock Woolly Adelgid in the Eastern United States Symposium* 329 333 Rutgers University

[bibr17] Flowers RW Salom SM Kok LT 2005 Competitive interactions among two specialist predators and a generalist predator of hemlock woolly adelgid, *Adelges tsugae* (Homoptera: Adelgidae) in the laboratory. *Environmental Entomology* 34 664 675

[bibr18] Flowers RW Salom SM Kok LT 2006 Competitive interactions among two specialist predators and a generalist predator of hemlock woolly adelgid, *Adelges tsugae* (Hemiptera: Adelgidae) in south-western Virginia. *Agriculture and Forest Entomology* 8 253 262

[bibr19] Furniss RL Carolin VM 1977 *Western Forest Insects*. USDA Forest Service Miscellaneous Publication, No. 1339

[bibr20] Gordon RD. 1985 The Coccinellidae (Coleoptera) of America north of Mexico. *Journal of the New York Entomological Society* 93 1 912

[bibr21] Grostal P Dicke M. 1999 Direct and indirect cues of predation risk influence behavior and reproduction of prey: A case for acarine interactions. *Behavioral Ecology* 10 422 427

[bibr22] Han BY Chen ZM 2002 Composition of volatiles from intact and tea-aphid damaged tea shoots and their allurement to several natural enemies of the tea aphid. *Journal of Applied Entomology* 126 497 500

[bibr23] Harmon JP Losey JE Ives AR 1998 The role of vision and color in the close proximity foraging behavior of four coccinellid species. *Oecologia* 115 287 292 10.1007/s004420050518 28308464

[bibr24] Havill NP Montgomery ME Yu G Shiyake S Caccone A 2006 Mitochondrial DNA from hemlock woolly adelgid (Hemiptera: Adelgidae) suggests cryptic speciation and pinpoints the source of the introduction to eastern North America. *Annals of the Entomological Society of America* 99 195 203

[bibr25] Hemptinne JL Lognay G Gauthier C Dixon AFG 2000 Role of surface chemical signals in egg cannibalism and intraguild predation in ladybirds (Coleoptera: Coccinellidae). *Chemoecology* 10 123 128

[bibr26] Hironori Y Katsuhiro S 1997 Cannablism and interspecific predation in two predatory ladybirds in relation to prey abundance in the field. *Entomophaga* 42 153 163

[bibr27] Koch RL 2003 The multicolored Asian lady beetle, *Harmonia axyridis*: A review of its biology, uses in biological control, and non-target impacts. *Journal of Insect Science* 3: 32 Available online: insectscience.org/3.32 10.1093/jis/3.1.32 PMC524671 15841248

[bibr28] Koch RL Hutchinson WD 2003 Phenology and blacklight trapping of the multicolored Asian lady beetle (Coleoptera: Coccinellidae) in a Minnesota agricultural landscape. *Journal of Entomological Science* 38 477 480

[bibr29] Lamana ML Miller JC 1996 Field observations on *Harmonia axyridis* Pallas (Coleoptera: Coccinellidae) in Oregon. *Biological Control* 6 232 237

[bibr30] Lamana ML Miller JC 1998 Temperature dependent development in an Oregon population of *Harmonia axyridis* (Coleoptera: Coccinellidae). *Environmental Entomology* 27 1001 1005

[bibr31] Lamb AB Salom SM Kok LT 2005a Survival and reproduction of *Laricobius nigrinus* Fender (Coleoptera: Derodontidae), a predator of hemlock woolly adelgid *Adelges tsugae* Annand (Homoptera: Adelgidae) in field cages. *Biological Control* 32 200 207

[bibr32] Lamb AB Salom SM Kok LT 2005b Guidelines for rearing *Laricobius nigrinus* Fender. Onken B Reardon R *Proceedings: Third Symposium on Hemlock Woolly Adelgid in the Eastern United States* 309 318 USDA Forest Service

[bibr33] Lamb AB Salom SM Kok LT 2006 Confined field release of *Laricobius nigrinus* (Coleoptera: Derodontidae), a predator of the hemlock woolly adelgid *Adelges tsugae* Annand (Homoptera: Adelgidae), in Virginia. *Canadian Journal of Forest Research* 36 369 375

[bibr34] Larock DR Ellington JJ 1996 An integrated pest management approach, emphasizing biological control, for pecan aphids. *Southwestern Entomologist* 21 153 167

[bibr35] Lu W Souphanya P Montgomery ME 2002 Descriptions of immature stages of *Scymnus (Neopullus) sinuanodulus* Yu and Yao (Coleoptera: Coccinellidae) with notes on life history. *The Coleopterists Bulletin* 56 127 141

[bibr36] Majerus ME 1994 *Ladybirds*. Harper Collins Publishers

[bibr37] Matsuka M Niijima K 1985 *Harmonia axyridis*. Singh P Moore RF *Handbook of Insect Rearing, Vol. I* Elsevier

[bibr38] Mausel DL 2004 Establishment and sampling of *Laricobius nigrinus* Reardon R Onken B *Proceedings: Hemlock Woolly Adelgid Biological Control Committee Meeting* 16 17 USDA Forest Service

[bibr39] Mausel DL 2005 Observations on fecundity and natural enemies of hemlock woolly adelgid, *Adelges tsugae* Annand (Hemiptera: Adelgidae) in Seattle, Washington. *The Pan-Pacific Entomologist* 81 97 98

[bibr40] Mayer M Chianese R Scudder T White J Vongpaseuth K Ward R 2002 Thirteen years of monitoring the hemlock woolly adelgid in New Jersey forests. Onken B Reardon R Lashomb J *Proceedings: Hemlock Woolly Adelgid in the Eastern United States Symposium* 50 60 Rutgers University

[bibr41] McClure MS 1987 Biology and control of hemlock woolly adelgid. *Connecticut Agricultural Experiment Station Bulletin* 851 3 9

[bibr42] McClure MS 1989 Evidence of a polymorphic life-cycle in the hemlock woolly adelgid, *Adelges tsugae* (Homoptera: Adelgidae). *Annals of the Entomological Society of America* 82 50 54

[bibr43] McClure MS 1991 Density-dependent feedback and population cycles in *Adelges tsugae* (Homoptera: Adelgidae). *Environmental Entomology* 19 36 43

[bibr44] McClure MS 1992 Effects of implanted and injected pesticides and fertilizers on the survival of *Adelges tsugae* (Homoptera: Adelgidae) and on the growth of *Tsuga canadensis*. *Journal of Economic Entomology* 85 468 472

[bibr45] McClure MS 1995 Using natural enemies from Japan to control hemlock woolly adelgid. *Frontiers in Plant Science* 47 5 7

[bibr46] McClure MS Cheah CASJ 1999 Reshaping the ecology of invading populations of hemlock woolly adelgid, *Adelges tsugae* (Homoptera: Adelgidae), in eastern North America. *Biological Invasions* 1 247 254

[bibr47] McClure MS Salom SM Shields KS 2001 *Hemlock woolly adelgid*. USDA Forest Service, FHTET 01–03.

[bibr48] Michaud JP 2002 Invasion of the Florida citrus ecosystem by *Harmonia axyridis* (Coleoptera: Coccinellidae) and asymmetric competition with a native species, *Cycloneda sanuinea*. *Environmental Entomology* 31 827 835

[bibr49] Michaud JP Grant AK 2004 Adaptive significance of sibling egg cannibalism in Coccinellidae: comparative evidence from three species. *Annals of the Entomological Society of America* 97 710 719

[bibr50] Montgomery ME Lyon SM 1996 Natural enemies of adelgids in North America: their prospect for biological control of *Adelges tsugae* (Homoptera: Adelgidae). Salom SM Tigner TC Reardon RC *Proceedings of the First Hemlock Woolly Adelgid Review* 89 102 USDA Forest Service

[bibr51] Musser FR Shelton AM 2003 Factors altering the temporal and within-plant distribution of coccinellids in corn and their impact on potential intraguild predation. *Environmental Entomology* 32 575 583

[bibr52] Nakamuta K. 1982 Switchover in searching behavior of *Coccinella septempunctata* L. (Coleoptera: Coccinellidae) caused by prey consumption. *Applied Entomology and Zoology* 17 501 506

[bibr53] Nakamuta K 1985 Mechanism of the switchover from extensive to area-concentrated search behaviour of the ladybird beetle *Coccinella septempunctata bruckii*. *Journal of Insect Physiology* 31 849 856

[bibr54] Nakamuta K 1987 Diel rhythmicity of prey-search activity and its predominance over starvation in the lady beetle *Coccinella septempunctata bruckii*. *Physiological Entomology* 12 91 98

[bibr55] Obata S 1986 Mechanisms of prey-finding in the aphidophagous ladybird beetle, *Harmonia axyridis* (Coleoptera: Coccinellidae). *Entomophaga* 31 301 311

[bibr56] Obata S 1997 The influence of aphids on the behaviour of adults of the ladybird beetle, *Harmonia axyridis* (Coleoptera: Coccinellidae). *Entomophaga* 24 103 106

[bibr57] Obata S Johki Y 1990 Distribution and behaviour of adult ladybird, *Harmonia axyridis* (Coleoptera: Coccinellidae), around aphid colonies. *Japanese Journal of Entomology* 58 839 845

[bibr58] Orwig DA Foster DR Mausel DL 2002 Landscape patterns of hemlock decline in New England due to the introduced hemlock woolly adelgid. *Journal of Biogeography* 29 1475 1487

[bibr59] Osawa N 1993 Population field studies of the aphidophagous ladybird beetle *Harmonia axyridis* (Coleoptera: Coccinellidae): life tables and key factor analysis. *Researches on Population Ecology* 35 335 348

[bibr60] Osawa N 2000 Population field studies of the aphidophagous ladybird beetle *Harmonia axyridis* (Coleoptera: Coccinellidae): resource tracking and population characterisitics. *Population Ecology* 42 115 127

[bibr61] Palmer D Sheppard J 2002 Mass rearing *Pseudoscymnus tsugae*, a predator of hemlock woolly adelgid, at the New Jersey Department of Agriculture: Challenges and lessons. Onken B Reardon R Lashomb J *Proceedings: Hemlock Woolly Adelgid in the Eastern United States Symposium* 214 220 Rutgers University

[bibr62] Parker BL Skinner M Gouli S Ashikaga T Teillon HB 1999 Low lethal temperature for hemlock woolly adelgid (Homoptera: Adelgidae). *Environmental Entomology* 28 1085 1091

[bibr63] Reid W Parker BL Skinner M Gouli S Teillon HB 2002 Insect-killing fungi for management of hemlock woolly adelgid: A review of progress. Onken B Reardon R Lashomb J *Proceedings: Hemlock Woolly Adelgid in the Eastern United States Symposium* 198 204 Rutgers University

[bibr64] Rhea RJ 1995 Preliminary results for the chemical control of hemlock woolly adelgid in ornamental and natural settings. Salom SM Tigner TC Reardon RC *The First Hemlock Woolly Adelgid Review* 113 125 USDA Forest Service

[bibr65] Rhoades MH 1996 Key to first and second instars of six species of Coccinellidae (Coleoptera) from alfalfa in southwest Virginia. *Journal of the New York Entomological Society* 104 83 88

[bibr66] Rosenheim JA Wilhoit LR Armer CA 1993 Influence of intraguild predation among generalist insect predators on the suppression of an herbivore population. *Oecologia* 96 439 449 10.1007/BF00317517 28313662

[bibr67] SAS Institute. 2001 *SAS/STAT User's Guide, Version 8.2.* SAS Institute www.sas.com.

[bibr68] Salom SM Sharov AA Mays WT Neal JW 2001 Evaluation of aestival diapause in hemlock woolly adelgid (Homoptera: Adelgidae). *Environmental Entomology* 30 877 882

[bibr69] Sasaji H 1977 Larval characters of Asian species of the genus *Harmonia* Mulsant. *Memoirs of the Faculty of Education, Fukui University, Series II* 27 1 17

[bibr70] Sasaji H McClure MS 1997 Description and distribution of *Pseudoscymnus tsugae* sp. nov. (Coleoptera: Coccinellidae), an important predator of hemlock woolly adelgid in Japan. *Annals of the Entomological Society of America* 90 563 568

[bibr71] Sato S Hironori Y Evans EW 2005 Dropping behavior of larvae of aphidophagous ladybirds and its effects on incidence of intraguild predation: interactions between the intraguild prey, *Adalia bipunctata* (L.) and *Coccinella septempunctata* (L.), and the intraguild predator, *Harmonia axyridis* Pallas. *Ecological Entomology* 30 220 224

[bibr72] Snyder WE Joseph SB Preziosi RF Moore AJ 2000 Nutritional benefits of cannibalism for the lady beetle *Harmonia axyridis* (Coleoptera: Coccinellidae) when prey quality is poor. *Environmental Entomology* 29 1173 1179

[bibr73] Scares AO Coderre D Schanderl H 2004 Dietary self-selection behavior by the adults of the aphidophagous lady beetle *Harmonia axyridis* (Coleoptera: Coccinellidae). *Journal of Animal Ecology* 73 478 486

[bibr74] Stathas GJ Eliopoulos PA Kontodimas DC Giannopapas J 2001 Parameters of reproductive activity in females of *Harmonia axyridis* (Coleoptera: Coccinellidae). *European Journal of Entomology* 98 547 549

[bibr75] Stoetzel MB 2002 History of the introduction of *Adelges tsugae* based on voucher specimens in the Smithsonian National Collection of Insects. Onken B Reardon R Lashomb J *Proceedings: Hemlock Woolly Adelgid in the Eastern United States Symposium* 12 Rutgers University

[bibr76] Takahashi K. 1987 Differences in opposition initiation and sites of lady beetle, *Coccinella septempunctata bruckii* Mulsant and *Harmonia axyridis Pallas* (Coleoptera: Coccinellidae) in the field. *Japanese Journal of Applied Entomology and Zoology* 31 253 254

[bibr77] Wagner JD Glover MD Moseley JB Moore AJ 1999 Heritability and fitness consequences of cannibalism in *Harmonia axyridis*. *Evolutionary Ecology Research* 1 375 378

[bibr78] Wallace MS Hain FP 2000 Field surveys and evaluation of native and established predators of the hemlock woolly adelgid (Homoptera: Adelgidae) in the southeastern United States. *Environmental Entomology* 29 638 644

[bibr79] Webb RE Frank JR Raupp MJ 2003 Eastern hemlock recovery from hemlock woolly adelgid damage following imidacloprid therapy. *Journal of Arboriculture* 29 298 302

[bibr80] Yasuda H Shinya K 1997 Cannibalism and interspecific predations of lady beetles in spring alfalfa fields. *Japanese Journal of Entomology* 57 199 203

[bibr81] Yasuda H Takagi T Kogi K 2000 Effects of conspecific and heterospecific larval tracks on the oviposition behavior of the the predatory ladybird, *Harmonia axyridis* (Coleoptera: Coccinellidae). *European Journal of Entomology* 97 551 553

[bibr82] Zar JH 1998 *Biostatistical Analysis**3^rd^ edition.* Prentice-Hall

[bibr83] Zilahi-Balogh GMG Kok LT Salom SM 2002 Host specificity of *Laricobius nigrinus* Fender (Coleoptera: Derodontidae), a potential biological control agent of the hemlock woolly adelgid, *Adelges tsugae* Annand (Homoptera: Adelgidae). *Biological Control* 24 192 198

[bibr84] Zilahi-Balogh GMG Salom SM Kok LT 2003c Development and reproductive biology of *Laricobius nignnus*, a potential biological control agent of *Adelges tsugae*. *BioControl* 48 293 306

[bibr85] Zilahi-Balogh GMG Humble LM Lamb AB Salom SM Kok LT 2003a Seasonal abundance and synchrony between *Laricobius nigrinus* (Coleoptera: Derodontidae) and its prey, the hemlock woolly adelgid (Homoptera: Adelgidae) in British Columbia. *Canadian Entomologist* 135 103 11

